# Manifestations of dark personality traits in work contexts: a comparison of sociodemographic variables between Spain and Chile

**DOI:** 10.3389/fpsyg.2025.1642904

**Published:** 2025-11-12

**Authors:** Carla Bueno de la Fuente, Raquel de la Fuente-Anuncibay, Delfín Ortega-Sánchez, Jerónimo González-Bernal

**Affiliations:** 1 Department of Educational Sciences, University of Burgos, Burgos, Spain; 2 Department of Specific Didactics, University of Burgos, Burgos, Spain; 3 Department of Health Sciences, University of Burgos, Burgos, Spain

**Keywords:** Dark Triad, personality, sociodemographic variables, organizational culture, cross-cultural differences

## Abstract

**Introduction/objective:**

The Dark Triad of personality (Machiavellianism, narcissism, and subclinical psychopathy) has garnered increasing attention in organizational settings due to its impact on workplace behavior and organizational climate. This study aimed to examine the expression of these traits in work environments in Chile and Spain, taking into account potential differences based on geographic-cultural background, age group, occupation, and gender.

**Method:**

A non-experimental cross-sectional design was employed with a sample of 173 employed individuals. The Dark Triad of Personality at Work (TOP) questionnaire was administered, assessing egocentric views of work (EGO), imposing attitudes (IMPO), and impulsive/disengaged styles (NCI).

**Results:**

Male participants scored significantly higher across the three theoretical dimensions and in the subscales of Authority Need and Impulsivity (η^2^ = 0.026–0.054), although all scores remained within normative ranges. Chilean participants stood out in Leadership Attribution and Risk-Seeking (η^2^ ≈ 0.03); older adults (64–80 years) exhibited greater feelings of superiority compared to middle-aged adults (42–52 years); and managerial positions were associated with moderate-to-high scores in Leadership Attribution (*T* ≈ 56; η^2^ = 0.090). No significant differences were found in the global factors based on country, age, or profession.

**Conclusion:**

Overall, variables such as gender, geographic background, and organizational hierarchy appear to modulate adaptive expressions of dark personality traits, without reaching dysfunctional levels.

## 1 Introduction

In recent years, the scientific community has shown growing interest in examining how dark personality traits (Machiavellianism, narcissism, and subclinical psychopathy) affect leadership performance and organizational dynamics. These traits, typically linked to manipulative behavior, egocentrism, and lack of empathy, may also confer certain adaptive advantages in highly competitive environments (Paulhus and Williams, 2002). In this regard, the so-called Dark Triad of personality has been associated with dysfunctional behaviors, such as the instrumentalization of others, lack of empathy, and the pursuit of personal gain through interpersonal exploitation (Aluja et al., 2022; Doerfler et al., 2021; Duradoni et al., 2023; Junca-Silva and Silva, 2023; Nguyen et al., 2021). Its presence in the workplace can significantly deteriorate the organizational climate, increasing conflict, reducing employee cooperation, and negatively affecting workers’ psychological well-being.

Specifically, Machiavellianism is defined by a strategic orientation toward personal goal achievement through manipulation and the use of others as instruments (Christie and Geis, 1970). Narcissism, in turn, is characterized by a grandiose self-image, an intense need for admiration, and a limited capacity for empathy (Morf and Rhodewalt, 2001). Subclinical psychopathy in work settings is associated with emotional insensitivity, impulsivity, and a tendency toward moderate antisocial behavior (Babiak and Hare, 2006).

Nevertheless, these traits do not invariably lead to negative outcomes in organizational settings. In certain contexts, such as those demanding high competitiveness or rapid decision-making, individuals with elevated scores in one or more of these traits may exhibit strategic advantages (Kaufmann et al., 2021; McLarty and Holt, 2019). For example, narcissism can be linked to high levels of self-confidence and charisma, qualities that, under specific conditions, may facilitate leadership, particularly when modulated by prosocial traits that foster cooperation and ethical integrity (Brunzel, 2021; Fatfouta, 2019). In this sense, the relationship between leadership and dark personality traits constitutes a complex paradigm. While these traits may be related to valuable competencies in demanding contexts—such as persuasiveness, stress resilience, or risk-taking—when dysregulated, they may trigger interpersonal conflicts, unethical organizational practices, and the degradation of the workplace climate.

Within the field of personality psychology, the Dark Triad has likewise garnered increasing attention as a cluster of traits with significant implications for interpersonal and social behavior. While these traits share common elements, such as manipulation, lack of empathy, and egocentric orientation, each exhibits distinct characteristics: Machiavellianism is linked to a calculating and manipulative disposition; narcissism to an inflated self-image and a sense of superiority; and subclinical psychopathy to impulsivity, emotional detachment, and lack of remorse. Despite their frequent joint examination, it is essential to analyze their interaction with other personality traits to more deeply understand their impact across diverse contexts.

The scientific literature suggests that the effects of Dark Triad traits on job performance do not operate in isolation, but rather depend significantly on their interaction with other personality components (Miao et al., 2019). Certain trait combinations may either mitigate or intensify their influence in organizational settings. For instance, a high level of conscientiousness, a trait from the Five-Factor Model, may moderate the expression of Machiavellian tendencies, leading to strategic behavior that does not compromise the ethical standards of the organization. This integrative perspective enables the identification of personality configurations that promote both high performance and prosocial adaptation, as well as those that may give rise to dysfunctional behaviors with negative consequences for both individual and collective well-being (Ellen et al., 2021; Kowalski et al., 2021; Musek and Grum, 2021; Rico-Bordera et al., 2023).

Moreover, other personality traits such as humility and kindness, though not explicitly included in the Five-Factor Model, have been shown to play a significant role in modulating the effects of the Dark Triad. These traits can act as protective factors against narcissistic, Machiavellian, or psychopathic behaviors by fostering more ethical, cooperative, and empathetic interactions in the workplace (Howard and Van Zandt, 2020; Hudson, 2023; Puthillam et al., 2020).

Beyond deficit-centered models, contemporary motivational research suggests that the expression of dark traits in achievement contexts can be channeled adaptively when the social environment nurtures individuals’ basic psychological needs. Drawing on Self-Determination Theory (SDT), recent evidence in the educational field shows that frustration versus support of these needs is systematically associated with maladaptive versus adaptive functioning, including the ability to persevere and self-regulate effort. In this vein, using a large adolescent sample, Trigueros and García-Mas (2025) demonstrated that need frustration predicts lower grit and resilience, as well as less self-determined forms of regulation. Importantly, their analyses support the role of *novelty* as a fourth basic need alongside autonomy, competence, and relatedness. Complementarily, Trigueros et al. (2025) highlighted that interpersonal need-supportive behaviors among peers (e.g., fostering a partner’s autonomy, reinforcing competence, and promoting relational bonds) are positively related to engagement, grit, resilience, and intrinsic motivation, whereas need-thwarting behaviors show the opposite pattern. Although these studies were conducted in physical education settings, their mechanisms are general: when individuals perceive opportunities for volition, efficacy, supportive relationships, and novelty, they show enhanced self-regulation and persistence in the face of adversity.

Transposed to organizational psychology, these findings offer a constructive and complementary lens for interpreting the *Dark Triad* in the workplace. First, a *need-supportive organizational climate*, characterized by autonomy-supportive leadership, competence scaffolding, relational integration, and opportunities for novelty and innovation, may moderate how agentic and risk-taking tendencies linked to narcissism or psychopathy are expressed, channeling them toward functional outcomes such as proactive problem-solving, creative initiative, or crisis decision-making, rather than interpersonal exploitation. Conversely, *need-thwarting climates*, marked by controlling supervision, feedback that undermines competence, social exclusion, or monotonous/repetitive tasks, are likely to exacerbate Machiavellian instrumentalism or impulsive, disengaged styles, with negative implications for ethics and team climate.

The evidence regarding novelty as a psychological need is particularly relevant in contexts that value innovation. The finding that novelty frustration co-occurs with lower resilience and grit (Trigueros and García-Mas, 2025) suggests a concrete design lever: structuring jobs that are not only autonomous and socially integrated, but also varied and stimulating. In parallel, peer dynamics, team norms that either support or thwart needs, mirror classroom findings: peer support predicts higher perseverance and wellbeing (Trigueros et al., 2025). Consequently, rather than treating dark traits as uniformly detrimental (or advantageous), an integrative approach should consider how cultural and organizational structures shape the motivational context in which these traits are expressed. This complementary perspective aligns with our focus on the *adaptive expressions* of dark traits and provides a theoretical rationale for examining how sociocultural and sociodemographic factors modulate their manifestation in the workplace and across countries.

Despite the marked increase in research on the Dark Triad in workplace settings, much of the empirical evidence has been derived from homogeneous samples or single-country studies, often without sufficiently accounting for cultural differences. However, cultural factors play a critical role in shaping the social acceptance and behavioral expression of specific personality traits. Likewise, age group, professional occupation, and gender can act as influential sociodemographic moderators of behaviors related to manipulation, impulsivity, or emotional insensitivity. This highlights the need for more diverse and culturally sensitive approaches in this area of research. In this regard, several authors have noted that impulsivity and other psychopathic traits have a significant genetic basis; nonetheless, their expression can vary substantially depending on the cultural context (Larsson et al., 2006). In line with this, Matsunaga et al. (2000), in a study involving Japanese participants, identified clinical similarities between individuals with bulimia nervosa and those with multiple impulsivity traits when compared to Western populations, suggesting the presence of cross-cultural commonalities in the expression of these traits.

With respect to gender, the literature has documented notable differences in the manifestation of traits associated with the Dark Triad, particularly impulsivity. Prior studies have found that men tend to exhibit higher levels of impulsivity in both action execution and decision-making (Nautiyal et al., 2016; Yarmush et al., 2016). Chen et al. (2022) similarly reported that male participants scored higher than females across all assessed dimensions of impulsivity. However, they also observed conflicting findings regarding the neural correlates of these traits. Specifically, women demonstrated stronger correlations than men between sensation-seeking and the volume of brain structures such as the insula, putamen, and inferior frontal gyrus, as well as between positive urgency and the volume of the cingulate cortex, insula, and inferior frontal gyrus.

Complementarily, Rochat (2022) has highlighted that empathy also shows significant variation across developmental stages, influenced by early socialization factors, structural and functional brain differences, and both genetic and hormonal components. While there is broad consensus regarding the higher levels of impulsivity typically observed in males, the underlying neural mechanisms appear to differ by gender, underscoring the importance of incorporating this variable into studies on impulsivity and its psychological and biological correlates (Chen et al., 2022; Eisenberg et al., 2007; Yarmush et al., 2016).

Although certain personality traits, such as impulsivity, have a substantial genetic basis, their expression and social acceptance are modulated by cultural and gender-related factors. This reality underscores the need for an integrative research approach that systematically considers these variables. The lack of comparative studies examining such interactions in distinct occupational contexts, such as those of Chile and Spain, constitutes a significant limitation in understanding the cultural, sociodemographic, and gender-specific nuances of organizational behavior. Moreover, this gap hinders the development of intervention strategies tailored to specific contextual realities.

Based on these considerations, the primary aim of the present study was to analyze potential differences in the dimensions of dark personality (Machiavellianism, narcissism, and psychopathy) in workplace settings in Chile and Spain, with particular attention to cultural, sociodemographic, and gender-based differences. From a methodological standpoint, the comparison between Spain and Chile minimizes translation bias through the use of instruments administered in the same language (*linguistic control*), thereby enhancing both construct and measurement validity (Arenas-Gaitán et al., 2011). Theoretically, the two countries exhibit distinct cultural profiles across classical dimensions (e.g., power distance, uncertainty avoidance, and masculinity/individualism), which may modulate the expression and social valuation of dark traits and related behaviors (Hofstede, 2011). From an applied perspective, their organizational systems and labor markets share sufficient proximity to ensure comparability, while also displaying differences that enable the identification of potential moderating effects of culture and organizational climate (*socio-organizational* var*iation*). In this line, the following research hypotheses have been formulated:

H_1a_ (sex-factors). Men will score higher than women on the three dimensions of the TOP (EGO, IMPO, NCI). This expectation is grounded in the seminal Dark Triad model (Paulhus and Williams, 2002), in meta-analytic evidence documenting sex differences in narcissism (Grijalva et al., 2015a), and in neurobehavioral findings showing greater impulsivity among males (Chen et al., 2022).

*H_1b_* (sex-subscales): Men will outperform women on the subscales of authority, superiority, hardness, impulsivity, and concealment, remaining within normative levels, consistent with the literature on impulsivity (Chen et al., 2022).

*H_2a_* (country-factors): No significant differences by country (Chile vs. Spain) will be observed in the overall dimensions, consistent with the notion that cultural modulations tend to emerge more distinctly in subtraits than in aggregated factors (Arenas-Gaitán et al., 2011).

*H_2b_* (country–subscales): The Chilean sample will score higher than the Spanish sample on the leadership attribution and risk-taking subscales, with means remaining within the normative range. This expectation aligns with cultural arguments concerning leadership and entrepreneurship norms (Hofstede, 2011), the salience of leadership in competitive contexts (Cohen, 2016), and evidence linking dark traits to risk-taking behaviors reported in Latin America (Jahanshahi et al., 2023).

*H_3_* (age-superiority subscale): Older adults (64–80) will exhibit higher scores on the superiority subscale than middle-aged adults (42–52), within normative levels.

*H_4_* (occupation-leadership attribution subscale): The group of executives/managers will show higher scores on the leadership attribution subscale compared to other occupational groups, in accordance with processes of self-selection and role demands.

*H_5_* (adaptive level): Across all comparisons (H_1_–H_4_), mean scores will remain within the normative ranges of the TOP, such that observed differences will reflect adaptive (non-dysfunctional) expressions of dark traits in the workplace.

## 2 Method

### 2.1 Participants

The sample consisted of a total of 173 workers selected through non-probability convenience sampling. This selection strategy was based on participants’ accessibility and availability, in order to obtain relevant comparative data across cultural contexts, sociodemographic characteristics, and gender in the workplace. Individuals under the age of 18 and those experiencing long-term unemployment were excluded from the study. The sample consisted of participants from Spain (*n* = 74; 42.8%) and Chile (*n* = 99; 57.2%). The gender distribution was 56 men (32.4%) and 117 women (67.6%). The mean age was 44.7 years (*SD* = 14.6; median = 45; range = 21–80; IQR = 31–57). Regarding occupational characteristics, most participants were technicians and professionals in scientific and intellectual fields (*n* = 123; 71.1%), followed by directors or managers (*n* = 31; 17.9%), and, to a lesser extent, accounting, administrative, and office service employees (*n* = 9; 5.2%). The presence of associate professionals and technical support staff (*n* = 4; 2.3%), elementary occupations (*n* = 4; 2.3%), and workers in personal services, hospitality, protection, and sales (*n* = 2; 1.2%) was marginal. Overall, the sample can be described as predominantly composed of highly qualified personnel, with a notable proportion occupying managerial positions and a smaller representation of administrative, support, and service profiles. From a hierarchical perspective, directors and managers represented approximately one-fifth of the total, whereas the substantial presence of technical and scientific profiles indicates a high level of qualification not necessarily associated with supervisory roles.

### 2.2 Instrument

The instrument used in this study was the TOP – *Dark Triad of Personality at Work*, developed by Schwarzinger and Schuler and adapted into Spanish by Arribas and Solar (2022). This tool is specifically designed to assess, in an integrated manner, the three core traits of the Dark Triad (narcissism, Machiavellianism, and psychopathy) in workplace contexts. The instrument has demonstrated adequate psychometric properties in previous studies, including both validity and reliability. In the context of the present research, satisfactory overall indices were obtained to support the internal consistency of the scales, as measured by Cronbach’s alpha (*α* = 0.939) and McDonald’s omega (*ω* = 0.933).

The development of the TOP was grounded in the empirical literature on the Dark Triad in organizational settings. The narcissism component was based on the Narcissistic Personality Inventory (NPI) developed by Raskin and Halls (1979) and its German adaptation by Schütz et al. (2004). The Machiavellianism dimension drew upon the classical work of Christie and Geis (1970). Finally, the psychopathy component was informed by Hare’s model (2003) and the Kiel Psychopathy Inventory (Kieler Psychopathie Inventar, KPI; Köhler et al., 2003).

The TOP consists of 60 items distributed across three factors corresponding to each of the Dark Triad traits, further subdivided into eleven specific subscales (Table 1). Each item describes attitudes or behaviors related to job performance, measured using a 7-point Likert scale ranging from *strongly disagree* to *strongly agree*.

**Table 1:** Factors/scales of the TOP.

Factor/Scale	Content
Egocentric work orientation (EGO)	Corresponds to the workplace expression of narcissism. Assesses an exaggerated self-esteem regarding one’s own importance, leadership capacity, and influence over others, as well as a preference for exercising authority over colleagues.
Leadership attribution (Lid)	Measures the personal conviction of possessing exceptional leadership abilities.
Confidence in persuasive power (Per)	Assesses the belief in an especially powerful capacity to convince and influence others.
Need to exercise authority (Aut)	Measures the enjoyment derived from exerting authority over others.
Risk seeking (Rie)	Evaluates the tendency to assume risks and to confront challenging tasks.
Sense of superiority (Sup)	Measures the perception of being superior to others and the belief that one’s own performance is outstanding.
Imposition-focused work attitude (IMPO)	Linked to the workplace manifestation of Machiavellianism. Assesses emotional hardness, distrust of others, and confidence in one’s own capacity to achieve work goals.
Coldness (Fri)	Assesses the absence of empathy in work contexts.
Skepticism (Esc)	Measures distrust regarding the intentions of co-workers.
Hardness (Dur)	Evaluates the belief that strength and harshness are necessary to attain professional objectives.
Uncommitted-impulsive work style (NCI)	Represents the workplace variant of subclinical psychopathy. Evaluates a disorganized, impulsive work style prone to irresponsible behaviors, including lying and rule-breaking.
Unreflectiveness (Irn)	Assesses spontaneity, lack of planning, and limited reflection during task execution.
Impulsivity (Imp)	Measures the lack of emotional control in the face of unforeseen events and external influences at work.
Truth concealment (Ocu)	Evaluates the willingness to lie or conceal information for strategic or professional gain.

Source: Schwarzinger and Schuler (2022).

### 2.3 Design and procedure

A descriptive, cross-sectional, and non-experimental study was conducted with a sample of participants from Chile and Spain. Data collection for the analysis of the influence of cultural, sociodemographic, and gender variables on the dimensions and subscales of the Dark Triad of personality in the workplace was carried out through the online administration of the *Dark Triad of Personality at Work* (TOP) questionnaire. Prior to participation, the objectives of the study were presented, and informed consent was obtained from all participants, ensuring adherence to ethical principles of anonymity, confidentiality, and voluntary participation. The estimated completion time for the questionnaire was approximately 15 min. Informed consent was recorded in each completed form.

The study was approved by the Bioethics Committee of the University of Burgos (ref. IO 16/2024). In accordance with Spanish Organic Law 15/1999 on the Protection of Personal Data, the information collected was limited exclusively to the data necessary for the proper execution of the study and did not include any personally identifiable information. Participation was entirely anonymous and voluntary.

### 2.4 Data analysis

Both descriptive and inferential statistical analyses were conducted. Descriptive measures included means and standard deviations for each of the variables considered. For group mean comparisons, one-way analysis of variance (ANOVA) was employed. Prior to conducting the ANOVA, the assumptions of normality and homogeneity of variances were verified using the Kolmogorov–Smirnov test (*p* ≥ 0.34) and Levene’s test (*p* > 0.79), respectively, for each theoretical dimension. Effect sizes were calculated using eta-squared (η^2^), and the statistical power of the tests was estimated to assess the practical significance of the observed results.

For the variables corresponding to the subscales within each TOP theoretical dimension, the assumption of normality was not satisfied; however, homogeneity of variances was confirmed. Given the sample size exceeding one hundred and the presence of homogeneous variances, ANOVA was retained as the analytical method. In this context, the robustness of the F statistic in the face of normality deviations was considered acceptable. Moreover, the central limit theorem was taken into account, supporting the approximation of the sampling distribution of the mean for similar sample sizes, as well as the preservation of the expected F distribution under the MSB/MSE ratio.

To control for Type I error arising from the multiplicity of contrasts (dimensions and 11 subscales), a combined analytical approach was adopted. For factors with more than two levels (age and occupational category), *post hoc* pairwise comparisons were conducted using a conservative Bonferroni adjustment, and adjusted *p*-values were reported when appropriate. In dichotomous contrasts (sex and country) and in analyses with moderately specified *a priori* hypotheses, emphasis was placed on interpreting effect size (η^2^) and observed power (1-*β*), rather than relying solely on significance testing.

To facilitate an initial straightforward interpretation of the levels of each dimension and to enable comparison with previous studies or similar samples, the factorial dimensions (EGO, IMPO, and NCI) were calculated using raw mean scores. However, their corresponding subscales were analyzed using standardized/transformed scores (PT) in order to support a normative interpretation of the results and to address the need to compare different indicators on a common scale through metric standardization. Accordingly, raw scores (PD) were transformed into PT scores, allowing the degree of presence of the assessed traits to be interpreted on a standardized scale with a mean (*M*) of 50 and a standard deviation (*SD*) of 10. These values represent a linear transformation of the raw totals and preserve the validity of the statistical contrasts, as the F, p, and η^2^ statistics are identical to those obtained using raw scores.

The practical advantage of using standardized scores lies in their ability to place all means on a common metric, thus facilitating normative interpretation and cross-scale comparison without altering the magnitude of effect sizes reported through η^2^. The interpretation of score ranges (Table 2) therefore supported the comparison of individual profiles with reference norms and enabled a more precise analysis of the impact of dark personality traits in the workplace context.

**Table 2:** Interpretative ranges of standardized scores (PT).

PT range	Interpretive level	General interpretation
< ~35	Very low	Markedly below the normative mean (minimal presence of the trait).
~35–39	Low	Noticeably below the normative mean (the trait is scarcely expressed).
~40–45	Medium-low (below average)	Slightly below the mean; the trait appears to a somewhat lesser extent than average.
~46–54	Medium (average)	At the normative group mean; typical or average level of the trait.
~55–60	Medium-high (above average)	Slightly above the mean; the trait is manifested somewhat more than in most people.
~61–65	High	Noticeably above the normative mean; high level of the trait.
> ~ 65	Very high	Markedly above the mean (very high presence of the trait).

All analyses were performed using IBM SPSS Statistics version 28.

## 3 Results

The results indicated no statistically significant differences in the three theoretical dimensions of the TOP based on the defined age groups, country of origin, or declared profession of the participants. However, gender-based differences were identified, with men scoring higher than women across all dimensions (*M* ♂ > *M* ♀ in EGO, IMPO, and NCI; *F*_(1, 171)_ = 4.91–9.73, *p* = 0.028–0.002, η^2^ = 0.028–0.054) (Table 3).

**Table 3:** Comparison of factor scores by gender.

TOP	♀ (*n* = 117)	♂ (*n* = 56)	*F*	gl	*p*	1-*β*	η^2^	CI 95% η^2^
	*M* (*SD*)	*M* (*SD*)						
EGO	3.26 (1.02)	3.63 (1.03)	4.91	1	0.028	0.95	0.028	[0.000; 0.093]
IMPO	2.99 (0.91)	3.34 (0.92)	5.46	1	0.021	0.95	0.031	[0.000; 0.098]
NCI	2.77 (0.71)	3.14 (0.73)	9.73	1	0.002	0.95	0.054	[0.000; 0.132]

Although both women and men tend, on average, to slightly disagree with narcissistic, Machiavellian, or psychopathic statements, men do so to a lesser extent, bringing their responses closer to the neutral midpoint (neutral point = 4). Considering a statistical power of 1-*β* ≈ 0.95, the risk of committing a Type II error is low. Moreover, the effect sizes range between 0.028 and 0.054, which, according to conventional benchmarks (0.01 = small, 0.06 = medium), fall within the small but consistent range. Despite the presence of real differences, gender accounts for only 2.8 to 5.4% of the variance in each trait, indicating that most variability is explained by factors other than gender. Consequently, the absolute difference between men and women is approximately three-tenths of a point, which likely reflects a gradient of expression rather than a clinically meaningful gap.

Men scored higher than women on five subscales: Need to Exercise Authority (T_Aut), Sense of Superiority (T_Sup), Harshness (T_Dur), Impulsivity (T_Imp), and Concealment of the Truth (T_Ocu) (Table 4). The first three belong to the EGO and IMPO dimensions and reflect a firm self-concept of leadership, a conviction of being superior to others, and a belief in strength as a means of achievement. The latter two correspond to the psychopathic (NCI) component, indicating emotional dysregulation and a willingness to lie for personal gain. Although the mean differences approach 3–4 points (*p* = 0.034–0.008), both groups remain within the 46–54 range, categorized as average or typical for the normative group. Thus, men tend to express a slightly greater inclination toward authority, superiority, and instrumental harshness, though without reaching high-risk levels that would compromise workplace coexistence.

**Table 4:** Comparison of scale scores by gender and country of origin.

TOP	♀ (*n* = 117)	♂ (*n* = 56)	F	gl	*p*	1-*β*	η^2^	CI 95% η^2^
	*M* (*SD*)	*M* (*SD*)						
T_Aut	48.8 (9.6)	52.3 (10.3)	4.56	1	0.034	0.57	0.026	[0.000; 0.090]
T_Sup	48.8 (9.2)	52.4 (11.1)	4.93	1	0.028	0.60	0.028	[0.000; 0.093]
T_Dur	48.6 (9.4)	52.8 (10.5)	7.19	1	0.008	0.76	0.040	[0.000; 0.113]
T_Imp	48.8 (10.0)	52.4 (9.5)	5.23	1	0.023	0.63	0.030	[0.000; 0,096]
T_Ocu	48.7 (9.3)	52.6 (10.7)	6.05	1	0.015	0.69	0.034	[0.000; 0,103]

	Chile (*n* = 99)	Spain (*n* = 74)						
	*M* (*DT*)	*M* (*DT*)						
T_Lid	51.6 (9.7)	47.9 (9.9)	6.08	1	0.015	0.69	0.034	[0.000; 0.103]
T_Rie	51.5 (9.7)	47.9 (10.0)	5.59	1	0.019	0.66	0.032	[0.000; 0.099]

Note. T_*: Transformed scores of the EGO, IMPO, and NCI scales.

Regarding geographic origin, the Chilean subsample scored higher than the Spanish subsample on the subscales *Leadership Attribution* (T_Lid) and *Risk-Seeking* (T_Rie), two core traits of work-oriented narcissism. The Chilean averages (≈ 51–52) are approximately one decile above the Spanish averages (≈ 48), remaining within the normative range and associated with modest effect sizes (η^2^ ≈ 0.03). These values suggest that the differences may reflect cultural nuances (e.g., appreciation of entrepreneurship or visible leadership in Latin American contexts) rather than substantial variations in the presence of dark traits.

The age-based analysis revealed a single significant contrast on the *Sense of Superiority* scale, which was higher in the oldest group (64–80 years) compared to the middle-aged group (42–52 years) (Table 5). While the older group reached a mean of 51.7 points and the middle-aged group fell to 47.2, both remained within the typical or average range for the trait. A life-course explanation may account for this difference: as individuals accumulate professional experience, senior workers tend to reaffirm their expertise, resulting in a more prominent self-perception relative to younger colleagues. However, the effect size was small to moderate (η^2^ = 0.056) and did not extend to other dimensions such as impulsivity or harshness, which generally tend to decline with age. Nonetheless, these findings should be interpreted with caution, as confirmation would require group sizes ≥ 30, allowing the Central Limit Theorem to ensure greater variance stability.

**Table 5:** Comparison of scale scores by age group.

TOP	20–30 (*n* = 42)	31–41 (*n* = 39)	42–52 (*n* = 36)	53–63 (*n* = 38)	64–80 (*n* = 18)	F	gl	*p*	1-*β*	η^2^	CI 95% η^2^
	*M* (*SD*)	*M* (*SD*)	*M* (*SD*)	*M* (*SD*)	*M* (*SD*)						
T_Sup	48.9 (8.9)	50.3 (9.5)	47.2 (8.0)	50.9 (10.2)	51.7 (14.2)	2.51	4	0.044*	0.72	0.056	[0.000; 0.119]

T_*: Transformed scores of the EGO, IMPO, and NCI scales. *p < 0.05 between age groups 42–52 and 64–80 years.

The professional category proved to be significant on the *Leadership Attribution* scale (T_Lid). Managerial and executive staff (Category 2) reached a mean score of 55.9 (classified as moderate-high), clearly surpassing both technical and scientific professionals (49.0) and all other occupational groups (42–50) (Table 6). The effect size (η^2^ = 0.090) was the largest observed in the study, and the statistical power (0.84) supports the reliability of this finding. According to these results, individuals in leadership positions report a higher self-perception of leadership, which is consistent both with the demands of their roles and with self-selection processes, whereby individuals with a stronger (adaptive) narcissistic orientation are more likely to pursue positions of power.

**Table 6:** Comparison of scale scores by profession.

TOP	1 (*n* = 123)	2 (*n* = 31)	3 (*n* = 9)	4 (*n* = 4)	5 (*n* = 4)	6 (*n* = 2)	F	gl	*p*	1-*β*	η^2^	CI 95% η^2^
	*M* (*SD*)	*M* (*SD*)	*M* (*SD*)	*M* (*SD*)	*M* (*SD*)	*M* (*SD*)						
T_Lid	49.0 (9.61)	55.9 (9.46)	49.0 (20.0)	46.7 (11.36)	42.1 (11.10)	42.4 (14.64)	3.32	5	0.007**	0.84	0.090	[0.000; 0.161]

T_*: Transformed scores of the EGO, IMPO, and NCI scales. 1 = Technicians and scientific/intellectual professionals, 2 = Executive and managerial staff, 3 = Office workers, 4 = Support technicians, 5 = Elementary occupations, 6 = Workers in food service, personal care, protection, and sales. **p < 0.01 between scientific/intellectual professionals (1) and executive/managerial staff (2).

No significant differences were found across professions in traits such as harshness, impulsivity, or emotional coldness. This supports the notion that the Dark Triad is not a defining feature of ordinary occupational life, except in its component of self-attributed leadership.

## 4 Discussion and conclusions

Culture plays a significant role in shaping how individuals express dark personality traits. According to Hofstede’s (2001) model of cultural dimensions, factors such as individualism versus collectivism may moderate the social acceptance and expression of egocentric, manipulative, or emotionally insensitive behaviors. In this regard, recent research has suggested that in more collectivist cultures such as Chile, manifestations of Machiavellianism tend to be less explicit or direct than in more individualistic cultures such as Spain (López et al., 2019).

In line with these cultural considerations, evidence suggests that the interplay between Dark Triad traits and certain contextual moderators may, in Chile, foster both leadership attribution and risk propensity. At the individual level, narcissism has been positively associated with the emergence of leadership (though not necessarily with its effectiveness), a circumstance that increases the likelihood that such individuals will be perceived and selected as leaders (Grijalva et al., 2015a; Grijalva et al., 2015b). In parallel, dark traits, particularly subclinical psychopathy, have been linked to heightened sensation-seeking and risk-taking behaviors, as well as to the perception that such behaviors can yield benefits in competitive environments (Kennedy et al., 2021). At the situational level, climates characterized by high organizational politics and low accountability tend to amplify and “reward” instrumental behaviors associated with the Dark Triad, facilitating their visibility and advancement (Cohen, 2016). Moreover, in Latin America, there is emerging evidence that certain productive contexts value adaptability and self-promotion, showing positive associations between dark traits and work behaviors related to risk-taking (Jahanshahi et al., 2023). In this regard, more charismatic or participatory leadership cultures, together with organizational settings perceived as ambiguous or politicized, may be catalyzing leadership attribution and risk-taking in Chile as compared to Spain.

Notwithstanding the above, empirical evidence indicates that dark traits are frequently associated with antisocial behaviors and unethical attitudes across diverse cultural contexts. For instance, these traits have been shown to predict academic dishonesty (Curtis, 2023) and ethically questionable consumer practices (Egan et al., 2014) in international samples, suggesting a relative universality in their behavioral impact. However, the specific expression of these traits may differ significantly depending on the cultural context. Brownell et al. (2023), for example, identified nonlinear relationships between levels of dark traits in business founders and organizational performance, indicating that moderate levels of such traits may be adaptive in certain entrepreneurial environments. These findings point to the possibility that prevailing norms and values in a given culture, particularly in business settings, can modulate whether these traits function as adaptive or dysfunctional.

The scientific literature underscores the complexity and multidimensionality of dark personality traits, as well as the need to consider them in interaction with cultural, sociodemographic, and gender variables (Hofstetter and Filsinger, 2023; Rogoza and Cieciuch, 2018). Although the effects of the Dark Triad demonstrate a certain degree of cross-cultural consistency, it is likely that social norms, shared values, and culturally embedded role expectations influence how these traits are expressed in specific contexts. Consequently, further cross-cultural research is needed to deepen our understanding of the intricate interactions between personality and culture.

At the same time, numerous studies have documented significant gender differences in the expression of dark traits. In general terms, men tend to score higher than women in Machiavellianism and subclinical psychopathy, whereas gender differences in narcissism have produced more mixed results (Grijalva et al., 2015a; Grijalva et al., 2015b). These disparities may be mediated by sociocultural factors, gender norms, and social expectations tied to traditional role patterns. In this regard, Miller et al. (2010) proposed the concept of the *Vulnerable Dark Triad* (VDT), which includes vulnerable narcissism, the secondary factor of psychopathy, and traits associated with borderline personality disorder. This framework suggests the existence of subtypes or differential profiles within the Dark Triad traits, whose expression may vary according to gender and other contextual factors.

The contrasts conducted in the present study reveal a specific pattern characterized by small-to-moderate effect sizes (η^2^ = 0.026–0.090) and statistical power values that only occasionally reach the optimal threshold (1-*β* = 0.57–0.84). Therefore, the practical relevance of the observed differences should be interpreted with caution. Given that most of the obtained scores fall within the normative average range, the identified differences appear to reflect sample characteristics related to role, such as being male, Chilean, older, or holding a managerial position, rather than pathological deviations in personality.

As a result, the findings outline a profile of the worker who, when occupying positions of authority or possessing greater professional experience, tends to perceive themselves as a competent leader, somewhat superior and willing to act decisively. However, extreme emotional coldness, interpersonal cynicism, and manipulative concealment do not show systematic increases across any of the variables examined.

### 4.1 Practical implications for organizational settings

In the domain of selection, the TOP should be used as a complementary, non-determinant indicator, aimed at identifying adaptive manifestations aligned with requirements of influence and decisional agility (e.g., higher leadership attribution and risk-taking), provided these coexist with low impulsivity and limited tendencies toward concealment. In our analysis, these differences were modest and within the normal range (PT ≈ 46–54), with Chilean means exceeding Spanish ones in leadership and risk (η^2^ ≈ 0.03), and slightly higher male scores in impulsivity and concealment, without reaching elevated risk levels. To preserve fairness, interpretation should rely on PT norms and multimethod decision processes (structured interviews and situational judgment tests, SJTs), which can provide incremental validity and enhance behavioral prediction, while avoiding rigid cut-off scores and controlling for potential adverse impact (Christian et al., 2010).

Regarding leadership development, it is advisable to channel the adaptive narcissistic self-confidence observed in managerial positions (moderate-to-high PT values in leadership attribution) toward responsible forms of influence through feedback, pressure-decision training, and accountability commitments, given that the largest differences are concentrated in this subscale rather than in extreme coldness, cynicism, or hardness. Finally, in the well-being and ethics domain, organizational “guardrails” are recommended (e.g., supervision of sensitive decisions, rotation of high-risk tasks, whistleblowing channels, and self-regulation training), accompanied by periodic monitoring when role or context changes occur.

### 4.2 Limitations and future research directions

The present study relied on non-probabilistic convenience sampling and employed a cross-sectional descriptive design. Both methodological decisions entail fundamental restrictions for external validity and causal inference. Indeed, the absence of random participant selection precludes assurance of population representativeness, meaning that observed differences may reflect sample-specific characteristics rather than those of the reference population. Likewise, the cross-sectional nature of the study prevents the establishment of temporal directionality or causal relationships among variables (McMillan and Schumacher, 2005). Consequently, the generalization of the results must be considered limited, and the identified cultural effects should be interpreted as exploratory, pending confirmation through more robust designs.

Similarly, the reduced size of certain subgroups (e.g., specific age ranges or professional categories) decreases statistical power and estimation stability, thereby increasing the probability of Type II errors and widening confidence intervals. Comparisons between subgroups should, therefore, be interpreted with caution. Additionally, the use of self-report measures may introduce response biases (such as social desirability and acquiescence), as well as discrepancies between reported and observed behavior.

Furthermore, the present study neither examined nor controlled for potential confounding variables, which may affect internal validity and weaken the causal interpretation of observed differences across sex, country, age groups, and professional categories. In particular, unmeasured factors such as job tenure, education level, or employment sector might have partially influenced the results. Since comparisons were conducted via ANOVA without covariate adjustment, it was not possible to statistically isolate these extraneous effects, and thus, the observed effect sizes should be considered conditional upon this omission. This caveat aligns with classical methodological perspectives, which emphasize that uncontrolled factors may introduce ambiguity in causal attribution (Díaz Narváez, 2009), as well as with recent methodological guidelines recommending the identification and inclusion of potential confounders when explanatory relationships are sought (Hinojosa et al., 2024).

Future research should aim to: (a) implement probabilistic sampling with adequate subgroup sizes to improve precision and external validity; (b) employ longitudinal designs to estimate temporal trajectories and strengthen internal validity; (c) adopt multilevel approaches capable of modeling individual and organizational variation simultaneously; (d) incorporate complementary measures (behavioral and administrative indicators) to enhance evidence convergence and reduce the potential impact of self-report bias; and (e) introduce relevant covariates (through ANCOVA/GLM, stratification, or propensity score methods). Moreover, interpretive caution should be maintained given the small observed effects, and future multinational studies should verify metric and scalar invariance to ensure valid cross-country comparisons (Putnick and Bornstein, 2016).

## Data Availability

The raw data supporting the conclusions of this article will be made available by the authors, without undue reservation.
